# A rare clinical image of Chiari II-associated meningomyelocele in a late preterm neonate

**DOI:** 10.11604/pamj.2025.52.135.48378

**Published:** 2025-12-03

**Authors:** Rutuja Sawalkar, Sharath Hullumani

**Affiliations:** 1 Department of Paediatrics Physiotherapy, Ravi Nair Physiotherapy College, Datta Meghe Institute of Higher Education and Research, Sawangi (Meghe), Wardha, Maharashtra, India

**Keywords:** Meningocele, terminal myelocystocele, lipomyelomeningocele

## Image in medicine

A 20-day-old female late-preterm neonate (34+4 weeks, birth weight: 920 g) born via lower segment cesarean section for breech presentation to a primigravida mother presented with a lumbosacral swelling noted at birth. The neonate cried immediately after delivery. On examination, a dorsally placed cystic swelling with exposed neural placode was observed, consistent with an open neural tube defect. Lower-limb tone was reduced, with poor spontaneous movements and absent anal reflex. No craniofacial anomalies were noted. Magnetic resonance imaging (MRI) of the spine and brain demonstrated a lumbosacral meningomyelocele with herniation of neural elements through a bony defect, along with features of Chiari II malformation, including downward displacement of cerebellar tonsils and a small posterior fossa. The three differential diagnoses included meningocele, terminal myelocystocele, and lipomyelomeningocele. These findings confirmed meningomyelocele with associated Chiari II malformation. Screening cranial ultrasound showed mild ventriculomegaly. The neonate was managed in the neonatal intensive care unit (NICU) with a sterile dressing of the lesion, prone positioning, infection prevention measures, and fluid/electrolyte monitoring. Neurosurgical repair of the defect was planned (or completed on the day after birth). Supportive physiotherapy included positioning, maintaining skin integrity, and monitoring lower-limb responses. Postoperatively, the neonate remained hemodynamically stable with no immediate surgical complications. Neurological status and head circumference are being monitored for potential development of hydrocephalus.

**Figure 1:**
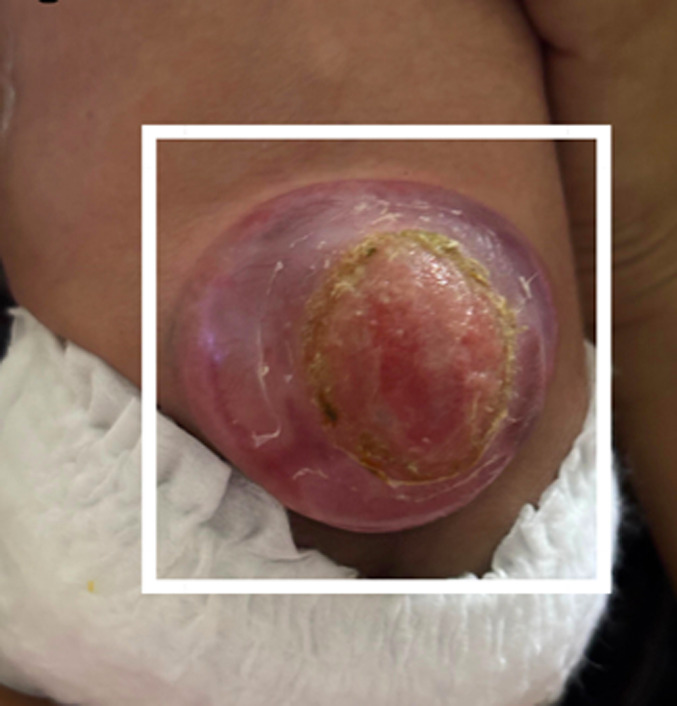
Chiari II-associated meningomyelocele

